# Hospital and Institutional News

**Published:** 1918-07-06

**Authors:** 


### HOSPITAL AND INSTITUTIONAL NEWS.

### DEATH OF A HOSPITAL CHAIRMAN.

On Saturday, June 29, there died at Cardiff, from heart failure, Mr. John P. Cadogan, the chairman of the executive committee of the Prince of Wales' Hospital for Limbless Sailors and Soldiers, Cardiff. Mr. Cadogan was the real founder of the hospital, in that, when the idea of such a hospital was first mooted in September, 1916, he at once gave a sum of £5,000, which covered the cost of the old mansion-house at Cardiff and the two adjoining houses in which the hospital is housed. From that time, Mr. Cadogan, as chairman of the executive committee, has done most useful work. His fatal illness lasted about a fortnight, and during that time visits were paid to him by the Rt. Hon. John Hodge, M.P. (Minister of Pensions), and Mrs. Lloyd George. Mr. Cadogan was head of the firm of Morgan and Cadogan, Ltd., coal shippers, Cardiff Docks, and director of two colliery companies. He was associated with the late Lord Rhondda in several business enterprises.

### INTER-ALLIED EXHIBITION OF AFTER-CARE METHODS.

The Inter-Allied Exhibition on the After-care of Disabled Men was opened at the City Hall, Cardiff, on Monday, June 24, by the Rt. Hon. John Hodge, M.P. (Minister of Pensions), and remained open during the whole week. This was the first provincial centre to be visited after the exhibition closed in London. The Lord Mayor of Cardiff (Alderman William Roberts, J.P.), chairman of the local committee, specially visited London at the beginning of June to put forward the claims of Cardiff, and himself undertook to raise the necessary guarantee to cover the heavy expenses involved. Mr. Hodge was supported at the opening ceremony by Sir A. Griffith-Boscawen, M.P. (Parliamentary Secretary to the Ministry of Pensions). The Cardiff visit has been in every way successful, and it is estimated that about 40,000 people attended during the week. One alteration made in the arrangement of the exhibition was to place in a separate room all exhibits relating to facial injuries.

### THE FILMS AND THE WELSH SECTION.

The cinematograph films were shown in another room, and about eight or nine shows were given each day. Probably the film which attracted most attention was that showing the development of kineplastic surgery under Dr. Putti, of Bologna. The films were explained by Dr. James Murphy (of the Italian Information Bureau), who also gave an exposition of the organisation in Italy of hospitals, training-centres, and pensions. A special feature of the exhibition was the Welsh section, consisting of working parties from the local training-centres: for tailoring, boot repairing, and clerical work at Cardiff; dental mechanics, watch, clock, and jewellery repairs, and motor mechanics at Swansea. Mr. A. B. Malden acted as organiser of the exhibition. The local secretaries were Mr. S. Auckland, secretary Joint Disablement Committee, South Wales and Monmouthshire; Mr. W. M. Davey, the Lord Mayor's secretary; and Mr. Gilbert D. Shepherd, secretary of the Prince of Wales' Hospital.

### THE STOCK EXCHANGE AND THE RED CROSS.

Ingenuity of appeals sometimes ends in the adoption of undesirable devices, and the *Morning Post* has uttered a word of warning in respect to the latest proposal which has been made to members of the Stock Exchange on behalf of the Red Cross. Members, it appears, have been invited not only to sell their own securities but to invite their clients to follow suit. It is pointed out that, if the latter course were widely adopted, the value of the stocks themselves might fall, since fancy prices would hardly be realised for shares and debentures, which, unlike vintages or works of art, do not necessarily increase in value with age, or acquire a *cachet* from long possession by a duke. It would be possible, no doubt, to organise a Stockbrokers' Day on which these gentlemen would be willing to give their commissions, or with their aid to issue an appeal to a section of the public which might otherwise remain unmoved. That is one thing. To endeavour to upset the ordinary stock markets is another.

### NOMENCLATURE OF HOSPITALS: AN ODD OMISSION.

In London we call hospitals after saints—Thomas, Bartholomew, John, and Elizabeth; or after localities—Middlesex, Wandsworth, Charing Cross; or after pious founders, like Guy. In the provinces you find a pious founder or two, but mostly it is the name of the town with "Infirmary" or "Hospital" tacked on to it, sometimes "Royal" being prefixed. But where will you find the name of a doctor given to a hospital, at least until the late Dr. Maudslay's (who, by the way, was a pious founder too, so that his is not a case in point) institution comes into its proper use? The interesting thing is that on the Continent they seem to think that a medical institution might sometimes be called after a famous medical man. There are the places that raffish genius Verlaine drifted in and out of, the hospitals Broussais and Bichât; there is the children's hospital Trousseau; there are hospitals called after Langenbeck and after Virchow. But in England, and in America too, to name a ward or a pavilion after a doctor is compliment enough. England, as Mr. Arnold Bennett (or was it Mr. Knoblock?) well remarked, has always known how to treat its men of genius. There is a certain national trait of indifference to things of the mind which it would be a good thing to eradicate in this twentieth century. When the shell-shock soldiers are all cured, and the Maudslay Mental Hospital gets going, it should not be left alone in its nomenclatural glory.

### VENEREAL DISEASES: A MEETING IN EDINBURGH.

The Lord Provost presided at a meeting in Edinburgh last month to consider the advisability of forming an Edinburgh branch of the National Council for Combating Venereal Diseases. The meeting resolved to form a branch, and founded it there and then. So much to the good, and no one would disagree with most that the speakers said. Sir Francis Champneys, Bart., said that if the community were moral it would be within measurable distance of stamping out venereal disease. Well, perhaps so, but is not the great mass of the community moral? It all depends on definition. On the whole, the community is moral despite the existence of a large percentage of habitually immoral persons in it. A great deal of venereal disease is due to infrequent lapses of persons moral, but suddenly exposed to temptation when in some condition that lessened their powers of resisting the Devil. Is it not important to remove the great tank of *infected* life represented by the prostitute from where it can infect those who succumb to the weakness of the flesh? Is it not important to remove those who infect the large proportion of women in our hospitals suffering from the results of gonorrhœa? He spoke of these women, but as innocent sufferers, not as women of the tank. Is the most urgent need to treat the diseased? It is a very urgent need, but is not the really urgent need to segregate the infected till they cease to be infectious. Till that is done venereal disease will scourge us, scourge equally the hardened sinner and the weakling who occasionally sins and deeply repents, and innocent wives. If a man has scarlet fever, small-pox, measles, typhoid fever, all comparatively innocuous diseases when compared with syphilis and gonorrhœa, no regard is paid to his feelings or conscience; he is "notified" and "segregated." Seldom, except in the case of a man who has refused vaccination, can it be suggested it was the man's fault he suffers from these diseases. Why should greater consideration be shown to those who have venereal diseases? The answer is obvious. The community, collectively, is afraid to know who of it and how many of it are affected. That is the bottom of it. That is why the disease cannot be made notifiable. That is why we shall continue to propagate it.

### PROPOSED MEMORIAL TO A HOSPITAL CHAIRMAN.

A largely-attended meeting has been called by the Mayor of Bournemouth to consider proposals for a town's memorial to the work of the late Dr. J. Roberts Thomson, Honorary Freeman of the Borough and first chairman of the Amalgamated Royal Victoria and West Hants Hospital. The Mayor referred to the great work of Dr. Roberts Thomson particularly in connection with the amalgamation of the Royal Victoria Hospital and the Royal Boscombe and West Hants Hospital, the success of which was largely due to him. Dr. Roberts Thomson's interests were many: education, Local Government work, and the Volunteer movement as well as hospital affairs obtained his support. He was a past president of the British Medical Association, and also of the Council of the British Medical Association, but it was felt nothing could be more appropriate than that the town's recognition of his services should be connected with the hospital for which he worked with such splendid results. The proposal was agreed to and a comprehensive committee has been formed to further the project and issue an appeal.

### NEW SECRETARY AT CHICHESTER.

The secretaryship of the Royal West Sussex Hospital, Chichester, vacant through the appointment of the present secretary, Mr. Tom B. Harrison, as general superintendent and secretary to the Devonshire Hospital, Buxton (The Hospital, June 15, p. 219), has been filled. At a meeting of the board of management, held on July 2, Mr. Alderman H. S. Aylmore, of East Street, Chichester, who has been a member of the board of management of the hospital for twelve years, was appointed secretary. The vacancy was advertised in last week's issue; the salary offered was £150, and previous hospital experience was stated to be essential. We understand that Mr. Aylmore has been in business, from which he is now retiring.

### HOSPITAL DEMONSTRATORS AND MILITARY SERVICE.

It was generally understood that men between the ages of forty-one and fifty-one engaged in highly skilled technical undertakings were not to be called upon to undergo medical examination under the new Military Service Act, and the Ministry of National Service issued instructions to their representatives containing a detailed list of the occupations which for the present were to be exempt. These instructions included education officers, teachers, and men engaged in public services, the nearest approach to anyone occupying a prominent position in a hospital or infirmary. In the view of the National Service representative neither of these classes cover lecturers and demonstrators in hospitals, so that Dr. M. S. Pembrey, of Guy's Hospital, was compelled to appeal at the Southwark Tribunal for the exemption of Mr. T. H. Evans, lecturer in biology (forty-two), and for Mr. R. F. Clark (forty-two), a demonstrator of dental mechanics, both in Grade 2. Happily the tribunal took the broad view, and granted them each six months' exemption.

### SERIOUS FIRE AT A V.A.D. HOSPITAL.

Oakwood V.A.D. Hospital, Rotherham, Yorkshire, was gutted by the recent fire, which was started in the roof by sparks from the kitchen chimney. There were ninety-seven wounded men in the building at the time, twenty of whom were cot cases. All were removed in time, and the contents of the hospital were mostly saved. Under the leadership of the matron, Miss Sinclair White, the convalescent men assisted the Rotherham Corporation Fire

Brigade, but the high wind and the low pressure of water at the height on which the hospital stood made the task a difficult one, and the damage is estimated at £5,000. Some of the wounded were removed to Sheffield, others to the Moorgate Military Hospital. The committee of the Oakwood Hospital, which belongs to Mr. Colin Smith, hope to secure another house where the work can be carried on.

### THE "FAILURE" OF SANATORIA.

Reporting on the suggestion by the recent conference of London local authorities with reference to the treatment of tuberculosis, Dr. Francis Stevens, the medical officer of health for Camberwell, accepts the conclusion that there should be a new authority to deal with insured and uninsured. He thinks that the medical officer of health is likely to have as good, and perhaps better, ideas of home conditions and circumstances of patients than the tuberculosis officer. As to the suggested segregation of advanced cases, he urges that what is wanted is an infirmary or sanatorium as a place which might be styled with some semblance of truth a home. There is no necessity for expensive buildings or elaboration of construction, for existing property could be well brought into use, and there are plenty of houses in London which could be used for this without detriment to their neighbours. He thinks that the advantages of the so-called educational treatment are over-estimated. Once a case reaches a sanatorium it should remain there till sufficiently improved, and that this work is more useful than "tinkering at a large number of cases." Here, as always, pitiful lack of sanatoria accommodation, to say nothing of a similar lack of trained, responsible medical superintendents, is the root, and the neglected root, of the "failure" of the recent campaign.

### SOME MISCONCEPTIONS EXPOSED.

Dr. G. Lissant Cox, tuberculosis medical officer for Lancashire, some time ago expressed the opinion that tuberculosis, especially when it affects the lungs ("consumption"), is an exceedingly difficult disease to cure or arrest unless it can be treated in the early stages. When the National Insurance Act drew special attention to tuberculosis, far too great emphasis was laid on the benefits of sanatorium treatment. The popular view was that any case, no matter in what stage, had only to be sent to a sanatorium and all would be well. The use of the term "sanatorium benefit," which really covers all kinds of benefit for tuberculosis cases, is unfortunate, and has undoubtedly assisted this erroneous belief. Because cases have been sent in the advanced stage of the disease to sanatoria instead of chest hospitals, and have not, of course, been "cured," public opinion is inclined to swing violently in the opposite direction and to say sanatorium treatment in general is useless. This view is equally wrong. Sanatorium treatment should be regarded merely as one kind of institutional treatment. Institutional treatment for tuberculosis in a complete scheme for dealing with the disease will be given in chest hospitals, general hospitals, sanatoria, and labour colonies, and for the following classes of patients:—(1) Early cases for a probable cure; (2) doubtful cases of tuberculosis, for purpose of diagnosis; (3) "education" cases, to receive treatment for a time in order that when they return home they may apply the general hygienic principles taught them at the sanatorium; and (4) isolation cases, which receive institutional treatment in order to prevent the spread of disease—these are usually advanced cases with home conditions which make satisfactory domiciliary treatment impossible. In any review of the curative results of schemes dealing with tuberculosis it is most important to remember that even at the present time the early cases applying for treatment are far outnumbered by the late and intermediate cases. In all measures dealing with tuberculosis emphasis should always be laid on the prevention rather than on the cure of the diseases. The eradication of the disease is perhaps the most difficult task yet attempted by preventive medicine, and the disease will only be eradicated slowly. The best results will be obtained if there be concentration (1) on the destruction of infected cattle; (2) on improvement in housing; (3) on education of the public as to the risks of the disease and how best to avoid them; (4) on a wider use by doctors of the services for diagnostic purposes of staffs appointed to carry out schemes for dealing with tuberculosis; and lastly on the isolation, as far as possible, of persons who are a serious danger to others.

### THE GUARDIANS OF THE MILK SUPPLY.

The excessive adulteration of milk in the metropolitan area is causing considerable anxiety to the public authorities, who, despite the fact that they are taking proceedings against the vendors, find that the percentage of adulteration is greatly on the increase. In the borough of Southwark during the last quarter the percentage of adulteration was found to be considerably greater than in any quarter since the opening of the Council's laboratory in 1902. In the opinion of the Southwark Borough Council this is an extremely serious matter, and the full facts are to be submitted to the magistrate by the Council's solicitor when next appearing to prosecute. The remedy lies not with the public authorities, but with the magistrates. There has been a tendency to fine delinquents lightly, and, consequently, they have grown in number.

### THE "WAAC," THE "WREN," AND THE MENTAL NURSE.

To supplement the list of the higher salaries granted by the London County Council to the women nursing staff in the mental hospitals (which was given in The Hospital of June 8), we may add to the £20 that a girl on entering the Asylum Service without previous experience now receives, the additional war bonus of 7s. a week, besides pay for food when off duty or away. An authoritative correspondent reminds us that this amounts to "considerably more than £40 a year, with no expenses of any kind." The reason for this increase is, flatly, that the Government, by its salaries to "Waacs" and "Wrens," has so raised the price of women's labour that it has set the pace for the mental hospitals too. Under the new scheme a young woman has only to wait three years, and maintain the not very arduous standard of conduct required, before becoming a charge nurse, at a considerable salary. Compared with the school nurse, who has to pay for board and lodging, the mental nurse is fortunate. As to the male staff, in peace-time a considerable number is resident, and during war a certain number must be resident too, in case of fire or emergency. It is the war bonus which has effected this improvement. A parallel, if extra-professional, advance is seen in the case of the private children's nurse, who, if qualified at one of the fashionable colleges or institutes, now regards £70 a year and a month's holiday, with the aid of an under-nurse, as a very modest demand. The registry offices are getting places for nurses, even without these semi-private qualifications, at £84 or even £100 per annum. If this class of woman can secure such pay, the case for the ordinary hospital nurse is a strong one, and her present salary is bound to rise soon. Now her chance is coming, she may be expected to make up for the long, lean years behind her.

### SANITARY SCIENCE IN SOUTH WALES.

JUDGING from the results of the recent examinations held at Cardiff by the Royal Sanitary Institute, sanitary science seems to be a difficult subject to study successfully in South Wales. Only three candidates sat in sanitary science as applied to building and public works, and all three failed to obtain a certificate. Seven sat for the inspector of nuisances examination, but only one passed. There were eleven candidates in the examination for women health visitors and school nurses, half of whom were ploughed. It is not only the Royal Colleges, therefore, which are determined to maintain a high standard.

### THE IMBECILE ATTENDANT.

IN view of the long hours and dissatisfied staff, the Infirmary Committee of the Portsmouth Board of Guardians has recommended the reorganisation of the male imbecile attendants. It has been decided, therefore, that they shall now work in eight-hour shifts for seven days a week, and that, apart from the head-attendant, an additional attendant shall be engaged, making the total seventeen. A staff of imbecile attendants is really an anachronism, notwithstanding the fact that it is still to be found at the present day.

### SIMPLE BUT EXPEDIENT.

IT must be the almost daily experience of hospital secretaries to receive from subscribers and friends announcements of the deaths of former practical sympathisers. A daughter will write to a hospital secretary and say that her mother has just passed away and that the deceased was a subscriber to the funds of the institution for many years. In such circumstances it is, of course, the custom of the hospital secretary to write a letter of condolence, but how many secretaries have imagination enough to keep in stock a supply of black-edged official paper for the purpose? We guess that very few follow this simple but praiseworthy course. Yet it must be a source of gratification to those who are in deep mourning to receive a letter of sympathy on notepaper that is in keeping with the message it conveys. This matter is not so small as it may appear, for we hear of at least one London institution where the deaths in the ranks of its annual subscribers number on an average no fewer than five a week.

### BOARD WAGES FOR SISTERS ON HOLIDAYS.

GLASGOW CORPORATION has asked its Special Committee on Wages to deal with a proposal by the Hospitals Committee to grant nurses who are provided with board and lodging at the hospitals a sum equivalent to the value of the board and lodging during holidays in addition to their salary.

### MUNICIPAL DENTISTRY.

THE Local Government Board is agreeable to municipal dentistry for the benefit of expectant or nursing mothers proposed by the St. Pancras Borough Council and the Local Government Board, and is prepared to pay a grant in connection with a central dental clinic, including the provision of new dentures in cases approved by the medical officer of the centre where the woman is not able to provide the dentures for herself.

### THIS WEEK'S DRUG MARKET.

A SLIGHT improvement in business is noticeable. The popular notion that our stocks of quinine are in danger of being exhausted in consequence of the demand for this drug by people who have, or fancy they have, influenza is, of course, incorrect; there is quite enough quinine in the country to satisfy the demands of the home trade, and the recent "rush" to the chemists has not affected the market. Phenacetin and phenazone still tend downwards in price. The allocation of a supply of formaldehyde for medical purposes is promised. Sugar of milk is dearer than ever. Sulphonal tends upwards in price. The demand for citric acid continues good. Bromides are quiet and unchanged. Salicylic acid continues in good supply. Gum guaiacum is very scarce, as also is calumba root. Only common qualities of castor oil are available.

### TO OUR READERS.

CONTRIBUTIONS are specially invited from any of our readers to these columns. They should deal with topical subjects and news. They must be authenticated for the information of the Editor only. The minimum payment if published is 5s. There is no hard-and-fast rule as to space, but notes of about twenty lines in length are preferred.

